# The Long and the Short of It: No Dietary Specialisation between Male and Female Western Sandpipers Despite Strong Bill Size Dimorphism

**DOI:** 10.1371/journal.pone.0079835

**Published:** 2013-11-15

**Authors:** Samantha E. Franks, Guillermo Fernández, David J. Hodkinson, T. Kurt Kyser, David B. Lank

**Affiliations:** 1 Department of Biological Sciences, Simon Fraser University, Burnaby, British Columbia, Canada; 2 Unidad Académica Mazatlán, Instituto de Ciencias del Mar y Limnología, Universidad Nacional Autónoma de México, Mazatlán, Sinaloa, México; 3 Aspect Ecology Ltd, West Court, Hardwick Business Park, Noral Way, Banbury, United Kingdom; 4 Department of Geological Sciences and Geological Engineering, Queen’s University, Kingston, Ontario, Canada; Norwegian Polar Institute, Norway

## Abstract

Many bird species show spatial or habitat segregation of the sexes during the non-breeding season. One potential ecological explanation is that differences in bill morphology favour foraging niche specialisation and segregation. Western sandpipers *Calidris mauri* have pronounced bill size dimorphism, with female bills averaging 15% longer than those of males. The sexes differ in foraging behaviour and exhibit partial latitudinal segregation during the non-breeding season, with males predominant in the north and females in the south. Niche specialisation at a local scale might account for this broad geographic pattern, and we investigated whether longer-billed females and shorter-billed males occupy different foraging niches at 16 sites across the non-breeding range. We used stable-nitrogen (δ^15^N) isotope analysis of whole blood to test for dietary specialisation according to bill length and sex. Stable-nitrogen isotope ratios increase with trophic level. We predicted that δ^15^N values would increase with bill length and would be higher for females, which use a greater proportion of foraging behaviour that targets higher-trophic level prey. We used stable-carbon (δ^13^C) isotope analysis to test for habitat segregation according to bill length and sex. Stable-carbon isotope ratios vary between marine- and freshwater-influenced habitats. We predicted that δ^13^C values would differ between males and females if the sexes segregate between habitat types. Using a model selection approach, we found little support for a relationship between δ^15^N and either bill length or sex. There was some indication, however, that more marine δ^13^C values occur with shorter bill lengths. Our findings provide little evidence that male and female western sandpipers exhibit dietary specialisation as a function of their bill size, but indicate that the sexes may segregate in different habitats according to bill length at some non-breeding sites. Potential ecological factors underlying habitat segregation between sexes include differences in preferred habitat type and predation risk.

## Introduction

Sex segregation is prevalent throughout the animal kingdom, and is widespread among avian taxa. Segregation of males and females during the non-breeding season can occur at spatial scales spanning broad geographic gradients to differences in microhabitat use [1]. Five hypotheses have been proposed to explain broad geographic gradients in the sex ratio of migratory bird species: the body size, dominance, arrival time, predation risk, and niche partitioning hypotheses [2]–[9]. Three of these hypotheses (dominance, predation risk, and niche partitioning) may also explain sex segregation at local scales.

Sexual size dimorphism is an ultimate driver of sex segregation, and size differences between males and females likely influence patterns of sex segregation through one or more of the aforementioned ecological mechanisms. Many shorebirds are sexually dimorphic in body size, with particularly pronounced dimorphism in feeding apparatus, notably bill size [10]. While sexual selection during the breeding season has been identified as an important factor influencing reverse sex size dimorphism in many shorebird species ([11], [12], but see [13]), niche specialisation as a consequence of bill size dimorphism has been proposed as a key factor influencing patterns of sex segregation in shorebirds during the non-breeding season [1], [14]–[20]. For example, differences in microhabitat use by male and female Icelandic black-tailed godwits *Limosa limosa islandica* have been attributed to niche specialisation, with prey distribution patterns and differences in the profitability of different prey types in relation to bill size driving sex segregation [19], [20].

In this study, we used isotopic measures of diet and habitat use to test a priori predictions about patterns of foraging niche differentiation in the western sandpiper *Calidris mauri* according to bill length and sex. Western sandpipers are sexually dimorphic in body size, with females the larger sex. While wing and tarsus length differ on average by about 5% between the sexes, western sandpipers exhibit pronounced bill size dimorphism: female bills average approximately 15% longer than those of males [21]–[23], but do not differ in either depth or curvature [24]. The sexes also differ in their proportional use of foraging modes, suggesting somewhat different foraging niches [7], [9], [25], [26]. Western sandpiper diet includes high-trophic level macrofaunal invertebrates such as large polychaetes, mid-trophic level meio- and macrofaunal invertebrates including crustaceans and bivalves, and low-trophic level biofilm, a surface matrix of microphytobenthos, organic detritus, and sediment in intertidal habitats [27]–[30]. Variation in bill length is associated with differences in the foraging mode used to access these various prey types. Short-billed males predominantly engage in pecking foraging behaviour, presumably used to feed on surface or near-surface prey, including small crustaceans such as copepods and cumaceans. Western sandpipers also graze on surface biofilm, and although the relationship between this foraging behaviour and bill length is unquantified, it is hypothesized to be more common among shorter-billed birds [28]. The long-billed females employ more probing behaviour, presumably to forage on more deeply buried prey such as large polychaetes [27]. The percentage of probing behaviour used by long-billed birds may be up to four times that of their shorter-billed counterparts [7], [9], [25], [26].

During the non-breeding season, western sandpipers are partially latitudinally segregated by sex. Towards the northern end of the wintering range (California), males comprise approximately 70–80% of populations, while the reverse occurs towards the southern end of the wintering range in Ecuador [31]. Foraging niche partitioning according to bill length has been proposed as a possible explanation for latitudinal sex segregation in western sandpipers, but the exact mechanism by which it acts is undetermined. Even within sexes, bill sizes are longer at more southern wintering sites, although this pattern is more pronounced in males than females ([7], [32], this study). This pattern further suggests a role for foraging niche specialisation in the distribution of non-breeding birds. Niche specialisation between males and females at a local scale is one potential mechanism that may drive broad patterns of latitudinal segregation (the local niche specialisation hypothesis). An alternative mechanistic explanation for latitudinal segregation that also invokes niche specialisation is that males and females distribute themselves according to habitat or dietary preference during the non-breeding season, but without niche partitioning at a local scale (the global niche preference hypothesis). A particular site’s sex ratio is then dependent on whether its habitat caters to male or female preferences, but there is no niche partitioning at the local level. While studies have found differences in foraging mode and habitat use according to bill size and sex at several wintering and migration sites [7], [9], [25], [26], [33], little work has directly examined niche segregation in relation to bill size at a local, site-specific level across the species’ range. In this study, we test the former mechanistic hypothesis and investigate whether niche specialisation occurs at a local scale as a function of bill length and sex in western sandpipers

We first re-examine latitudinal clines in western sandpiper bill morphology at a broad geographic scale and then investigate potential niche segregation at the local scale across the western sandpiper’s non-breeding range using stable-nitrogen and -carbon isotope analysis to infer diet and habitat use. Stable-nitrogen isotope analysis reflects the relative trophic level at which an individual is foraging, as the ratio of ^15^N/^14^N (δ^15^N) becomes enriched (higher) by 3–4 ‰ with increasing trophic level [34]. At a migratory stopover site, measured δ^15^N values of potential western sandpiper prey varied predictably in relation to trophic level, with that of biofilm measured as 6 ‰, that of small invertebrates, including bivalves, small polychaetes, and cumaceans, estimated as 8–9 ‰, and that of large polychaetes measured as 12 ‰ [29]. Stable-carbon isotope analysis reflects differences between terrestrial freshwater and marine carbon sources, with the ratio of ^13^C/^12^C (δ^13^C) becoming more enriched (higher) moving from freshwater to marine ecosystems [35]. Stable-carbon isotope values may reflect differential use of habitats influenced by marine carbon sources (e.g. open tidal mudflats) versus those more influenced by terrestrial freshwater (e.g. estuaries or freshwater marshes). Male and female western sandpipers in western Mexico showed differential use of marine- (brackish flats, male-biased sex ratio) versus freshwater- (cattail marsh, even sex ratio) influenced habitats [33].

We hypothesised that local niche segregation in western sandpipers might occur through two pathways, which are not necessarily mutually exclusive: 1) dietary specialisation on different prey types; or 2) habitat segregation. We predicted that if western sandpipers specialise on different prey according to bill length, then differences in bill morphology, which covary with foraging mode, should also influence trophic level and therefore δ^15^N values. Probing behaviour has been assumed to indicate foraging on polychaetes, a higher-trophic level prey, while pecking has been linked to foraging on small crustaceans, a mid-trophic level prey [27], [29], and possibly biofilm [28]. We therefore predicted that use of probing behaviour would correspond to feeding at a higher trophic level than using either pecking or grazing behaviour, and that δ^15^N values would increase with bill length and be higher in females compared to males.

At a non-breeding site in Mexico, males predominate in more marine habitats and females tend to use habitats influenced by freshwater input [33]. If this is a general pattern, we predicted that δ^13^C values would vary over a range of sites according to sex and/or bill length, being higher in males, which use more marine habitats.

## Materials and Methods

### Ethics statement

Capture, handling, and marking procedures were approved by the Simon Fraser University Animal Care Committee (#873B-08). All federal permits for the collection of blood were issued by Environment Canada, the U.S. Fish and Wildlife Service, México’s Secretaría de Medio Ambiente y Recursos Naturales, and Panama’s Autoridad Nacional del Ambiente.

### Sample collection

We captured 1051 western sandpipers at 16 wintering sites between mid-November and mid-February of 2008-09 (Table 1). Capture habitat varied among sites, and included intertidal mudflats, brackish lagoons, salt ponds, salt marshes, and freshwater marshes. We captured sandpipers using mist nets at one to several locations within each site, banded individuals, and measured exposed culmen (upper bill), tarsus, and wing length. Sex was assigned using culmen length [21], and birds were aged in the field as either adult (hatched at least two summers ago) or young (hatched the previous summer) by examining the edging colour of inner median coverts and the degree of flight feather wear [36], [37]. Of the 1051 captured birds, 1019 could be sexed using bill length criteria (females  =  467, males  =  552). We collected blood samples from western sandpipers by pricking the brachial vein and using a non-heparinized capillary tube to passively collect approximately 60 µL of blood. Within each sex, we randomly selected up to 5 blood samples from each site to analyse stable isotope ratios for a total of 124 samples. We analysed whole blood, which has a turnover rate of about 4 weeks in shorebirds [38], so that we could obtain a relatively long-term average representation of an individual’s winter diet and habitat use. Adult western sandpipers are generally well established at wintering sites by mid-October, while young individuals become established by early November. The majority of sampled birds in November were adults, and our interpretations assume that individuals had been established at their wintering site for at least one month prior to capture.

**Table 1:** Location, mean bill length, mean δ^15^N and δ^13^C blood values, and habitat type for each sampling site.

Site	Latitude	Longitude	Bill length mean ± SD		δ^15^N mean ± SD	δ^13^C mean ± SD *	Habitat
			Males	Females			
Salinas, Ecuador	–2.2	–80.8	21.2 (1)	26.8±1.0 (7)	12.1±1.4 (4)	–12.9±1.7	salt ponds
Costa del Este, Panama	9.0	–79.5	22.8±1.1 (16)	26.4±0.9 (59)	10.2±0.6 (8)	–15.8±0.7	marine-tidal
Cabo Rojo NWR, Puerto Rico	18.0	–67.2	22.6±0.8 (10)	27.0 (1)	6.1±0.8 (6)	–13.0±0.6	lagoon/salt ponds
Ría Lagartos, Yucatan, México	21.6	–88.0	23.1±0.8 (31)	27.1±1.1 (70)	7.4±0.4 (10)	–16.7±1.1	lagoon/salt ponds
Río Maximo & Tunas de Zaza, Cuba	21.7	–77.5	22.4±1.1 (23)	26.9±1.1 (37)	-	-	-
Laguna Huizache-Caimanero, Sinaloa, México	23.0	–106.0	23.0±0.9 (26)	27.0±0.9 (89)	8.2±0.7 (9)	–13.9±0.7	saltmarsh/lagoon
Ensenada de La Paz, Baja California Sur, México	24.1	–110.4	22.5±0.9 (87)	26.7±1.1 (21)	6.5±0.4 (10)	–6.9±0.7	marine-tidal
Ensenada de Pabellones, Sinaloa, México	24.5	–107.5	23.0±0.7 (30)	26.5±1.1 (27)	6.7±1.2 (7)	–13.2±2.4	saltmarsh/lagoon
Bahía de Santa María, Sinaloa, México	24.9	–107.9	22.7±0.8 (43)	26.4±0.6 (27)	9.2±1.1 (9)	–14.6±0.8	lagoon/salt ponds
Laguna Atascosa NWR, Texas	26.3	–97.4	23.4±0.4 (5)	26.9±1.1 (12)	8.6±1.0 (9)	–13.2±1.9	lagoon
Laguna Ojo de Liebre-Guerrero Negro, Baja California Sur, México	27.6	–114.1	22.6±0.9 (114)	26.5±1.0 (55)	6.8±0.5 (10)	–11.4±1.2	lagoon/salt ponds
St Marks NWR, Florida, USA	30.1	–84.2	22.2 (1)	25.9±0.3 (3)	7.4±0.5 (2)	–18.5±0.4	saltmarsh
Estero de Punta Banda, Baja California, México	31.7	–116.6	22.4±1.0 (18)	-	11.6±2.9 (6)	–13.1±1.8	marine-tidal
Alto Golfo de California y Delta del Río Colorado, Sonora, México	32.0	–114.8	22.1±0.9 (88)	26.4±0.9 (47)	8.1±1.3 (10)	–15.1±1.0	freshwater marsh
Yawkey Wildlife Center, South Carolina, USA	33.2	–79.2	22.7±0.7 (5)	26.7 (1)	8.8±0.4 (4)	–16.7±0.1	saltmarsh
San Francisco Bay, California, USA	38.1	–122.4	22.2±0.9 (42)	25.7±0.6 (8)	16.9±3.7 (10)	–15.2±1.3	lagoon/salt ponds
Humboldt Bay, California, USA	40.8	–124.1	22.2±0.9 (12)	26.3±0.6 (3)	14.1±0.4 (10)	–11.9±0.4	lagoon

Sample sizes are in parentheses. Habitat types of each site are general classifications of the locations in which birds were observed and captured.

* Sample sizes are the same as for δ^15^N values.

Freshwater marsh  =  no contribution of brackish or salt water from marine sources.

Lagoon  =  a shallow body of salt or brackish water where tidal turnover is low.

Salt ponds  =  artificial salt evaporation ponds.

Saltmarsh  =  vegetated marsh regularly flooded by salt or brackish water.

Marine-tidal  =  expansive tidal mudflats inundated daily.

### Isotope analysis

We analysed the majority of blood samples at the Queen’s Facility for Isotope Research in Kingston, Ontario, Canada between October 2009 and January 2010. Samples from the Yucatán, México, were analysed in December 2012. Samples were dried in a vacuum centrifuge and crushed into a powder. 0.2 – 0.4 mg of powder was loaded into tin capsules, converted to gas in an oxidation/reduction furnace (Costech ECS 4010 elemental analyser), and introduced on-line to an isotope ratio mass spectrometer (Delta^Plus^ XP). Stable isotope ratios are reported in delta (δ) notation in per mil (‰) units, where δX  =  ((R_sample_/R_standard_) - 1) x 1000. For nitrogen (δ^15^N), R  =  ^15^N/^14^N and R_standard_ is air, and for carbon (δ^13^C), R  =  ^13^C/^12^C and R_standard_ is a carbonate, V-PDB.

For every 50 nitrogen samples, we ran two laboratory standards (mean ± SD): domestic chicken (*Gallus gallus*) blood (δ^15^N  =  3.9±0.3 ‰, n  =  29) or silver nitrate ‘eil62’ (δ^15^N  =  16.8±0.2 ‰, n  =  4). Within each run, we also ran duplicates from the same individual, which produced a difference (mean ± SD) of 0.32±0.52 ‰ (n  =  12) for nitrogen samples and 0.14±0.13 ‰ (n  =  12) for carbon.

### Data analysis

We examined the relationship between bill length and latitude for each sex separately using a general linear model and least squares regression. We separated the sexes to be able to make sex-specific statements about the ability of latitude to explain variation in bill length. We used general linear mixed effects models and an information theoretic approach to model selection to compare competing hypotheses regarding the relationships of stable-nitrogen and -carbon isotope values with bill length and sex [39]. We included site as a random effect to account for differences in the baseline isotope values of primary productivity between sites. Before conducting our analysis, we removed two observations with extreme δ^15^N values, under the assumption that these were identifiable as individuals that had immigrated from other wintering sites. This left us with a sample size of 122 individuals. For analysis, bill length values were centred around the mean. δ^15^N and δ^13^C are frequently correlated [40], and although we examined the relationship between each isotopic composition and bill length and sex in two separate analyses, we first tested for correlation between the two isotopic compositions. δ^15^N and δ^13^C were only weakly correlated in our dataset (r = –0.22).

We used an information theoretic approach to model selection to determine the appropriate random effects structure for the relationship between isotope values and bill length: a random intercept (intercept allowed to vary randomly by site) or a random intercept and slope (intercept and slope of isotope vs. bill length relationship allowed to vary randomly by site). We used the R package *lme4* and restricted maximum likelihood estimation to fit mixed effects models with the same fixed effects structure (the most parameterised model below, Bill length x Sex), but different random effects [41]. We assessed the relationship between isotopes and bill length based on models using the optimal random effects structure: for δ^15^N, this was a random intercept and slope effect, while for δ^13^C, a random intercept only.

We wanted to explore whether niche segregation in western sandpipers occurs as a function of sex per se, or whether bill length dimorphism mediates differences in diet or habitat use between males and females. We included the following in our candidate model set: 1) bill length, which predicts differences in diet and habitat as a function of bill length alone; 2) sex, which predicts differences in diet and habitat as a function of sex alone; 3) bill length + sex, which predicts that diet and habitat might differ between the sexes, but within each sex, will vary according to bill length similarly; 4) bill length x sex, which predicts that diet and habitat is different for males and females and within each sex, varies differently according to bill length; and 5) a null model, which predicts that isotope values are the same across bill lengths and between males and females.

We specified no within-group correlations and used the maximum likelihood method to compare different fixed effect model structures. For each candidate model, Akaike’s Information Criterion corrected for small sample size (AIC_c_) was calculated from the maximum likelihood deviance (deviance  = –2 x log-likelihood). Models were ranked according to their ΔAIC_c_ score, calculated as the difference between a model’s AIC_c_ value and that of the best-supported model in the candidate set. The support for each model given by the data was evaluated using Akaike weight (AIC_c_ *w*), which represents the probability of the model, given the data, in relation to all other models in the candidate set. Parameter likelihoods and weighted parameter estimates for each explanatory variable were calculated to assess an individual variable’s relative importance within the candidate model set. All analyses were conducted in R [42]. The package *AICcmodavg* was used to calculate AIC_c_ values and produce model selection results [43].

## Results

**Figure 1:**
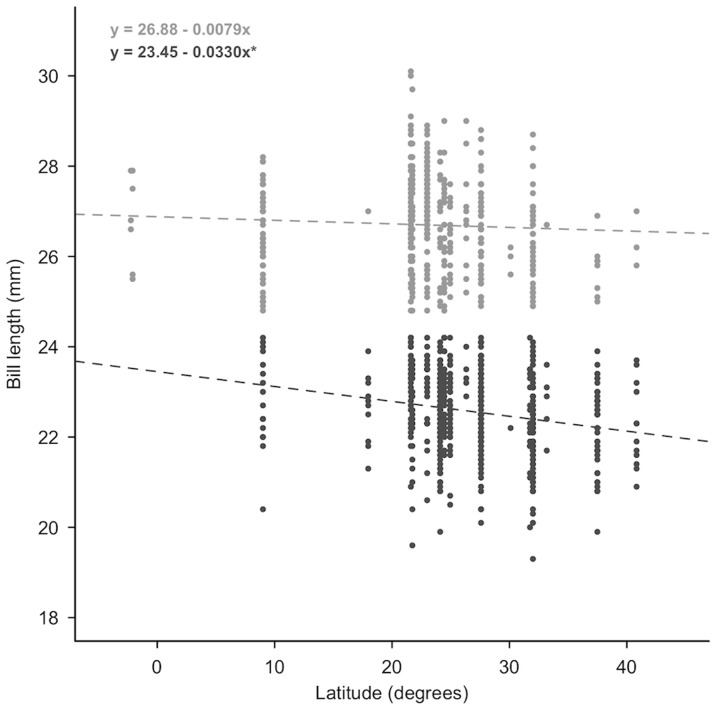
Relationship of bill length with wintering latitude by sex. The relationship of bill length with latitude for male (dark grey) and female (light grey) western sandpipers sampled at wintering sites between November 2008 and February 2009 (n = 1019). Fitted lines were calculated from linear regression parameter estimates, and the asterisk (*) denotes a significant relationship.

Bill lengths were significantly longer towards southern latitudes in males (Fig. 1, F_1,549_ = 25.1, p < 0.001, adjusted R^2^ = 0.042), but not in females (F_1,465_ = 1.45, p = 0.23, adjusted R^2^ = 0.001 ). Latitude, however, explained very little of the overall variation in bill lengths, even for males.

### δ^15^N

**Figure 2:**
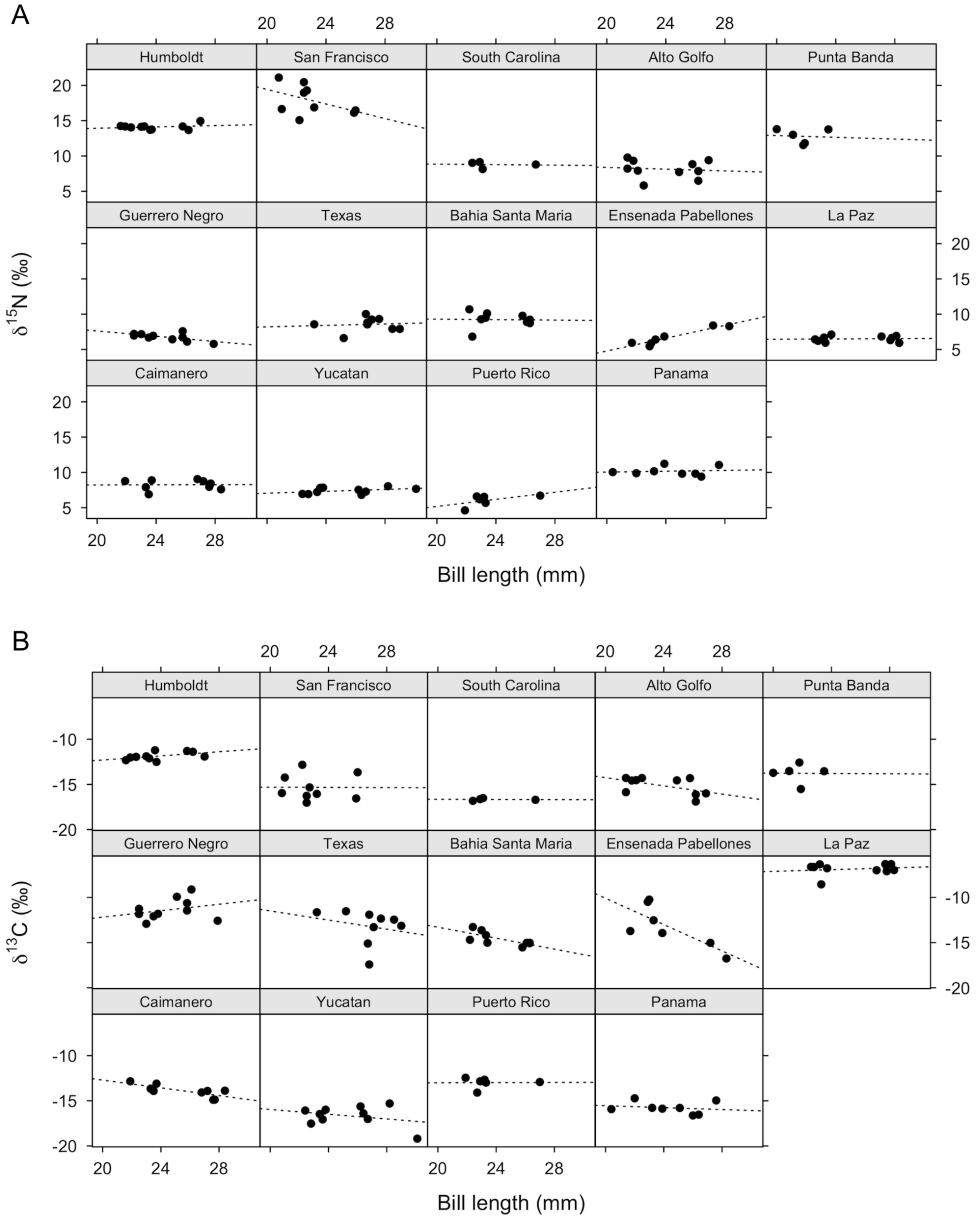
Relationship of a) δ^15^N and b) δ^13^C with bill length. All data (n = 122) were included in the model selection analysis, but only wintering sites with a total n > 4 are shown. Dotted lines represent the fitted linear regression line at each site. Sites are ordered latitudinally from north to south from top to bottom, and left to right.

**Figure 3:**
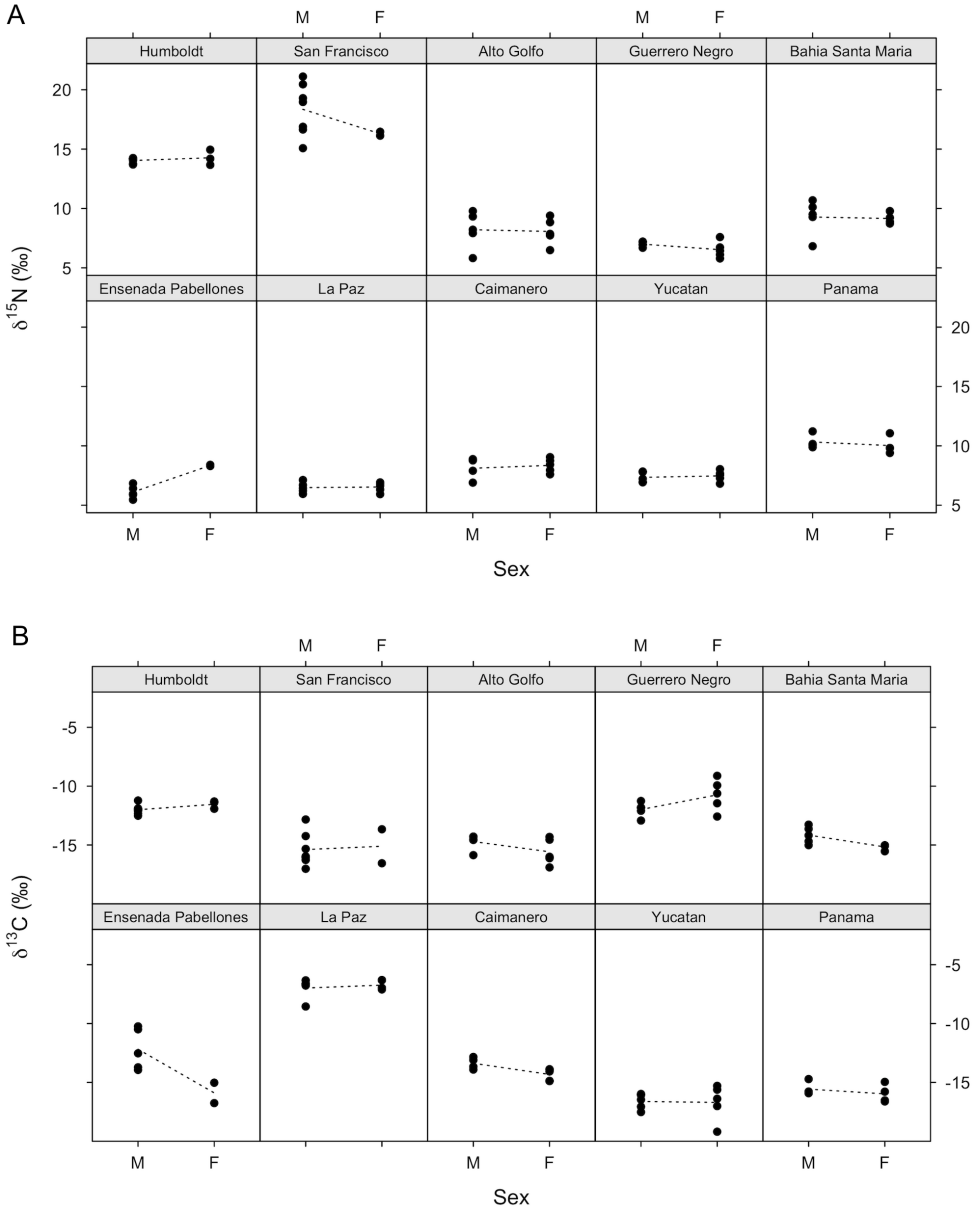
Relationship of a) δ^15^N and b) δ^13^C with sex. All data (n = 122) were included in the model selection analysis, but only wintering sites with n of each sex > 2 are shown. Dotted lines connect mean values. Sites are ordered latitudinally from north to south from top to bottom, and left to right.

The slope of the relationship between δ^15^N values measured from the blood of western sandpipers and bill length varied among sites (Fig. 2a; AIC_c_ *w* of the random intercept + slope model  =  0.68). Slopes were positive at some sites, negative at others, and at some sites there appeared to be no relationship. Similarly, there was no consistent difference in male and female δ^15^N values across sites (Fig. 3a). Model selection results indicated that neither bill length nor sex had any impact on δ^15^N isotope values (Table 2). The null model was the best supported, receiving nearly 50% of the weight and twice as much as the next most supported model, which included only an effect of sex. Both bill length and sex had very low parameter likelihoods, and the unconditional standard error of their weighted parameter estimates included zero (Table 3).

**Table 2:** Model selection results for candidate general linear mixed models used in the evaluation of the relationship between δ^15^N and δ^13^C values of western sandpiper blood (the response), bill length, and sex.

Isotope	Model	K	Deviance	AICc	ΔAICc	AIC_c_ w
δ^15^N	Intercept	5	404.85	415.37	0.00	0.52
	Sex	6	404.56	417.29	1.92	0.20
	Bill length	6	404.73	417.46	2.09	0.19
	Bill length+Sex	7	404.50	419.48	4.11	0.07
	Bill length*Sex	8	404.49	421.76	6.39	0.02
δ^13^C	Bill length	4	434.47	442.81	0.00	0.41
	Bill length+Sex	5	433.26	443.78	0.97	0.25
	Bill length*Sex	6	432.20	444.93	2.12	0.14
	Sex	4	437.03	445.37	2.56	0.11
	Intercept	3	439.79	445.99	3.18	0.08

Site was included as a random effect: all δ^15^N models included a random intercept and slope (by bill length for each site) effect, while δ^13^C models included only a random intercept. The number of parameters (K) includes a parameter for the intercept, variance of the random intercept, the residual variance, and also for the variance of the random slope, and the correlation between the estimated variances of the random components (if included in the model). Deviance is equal to -2 x log-likelihood and was used to calculate AIC_c_ (Akaike’s Information Criterion corrected for small sample size). Competing models were ranked according to ΔAIC­_c_ and Akaike weight (AIC_c_ *w*). The number of observations used for all models was 122.

**Table 3:** Parameter likelihoods and weighted parameter estimates for each explanatory variable included in the candidate model set.

Isotope	Explanatory variable	Parameter likelihood	Weighted parameter estimate	Unconditional SE	Lower 95% CI	Upper 95% CI
δ^15^N	Intercept	1.00	9.30	1.1	7.05	11.56
	Bill length	0.27	0.00	0.0	–0.05	0.04
	Sex	0.29	0.06	0.2	–0.39	0.51
	Bill length*Sex	0.02	0.00	0.0	–0.01	0.01
δ^13^C	Intercept	1.00	–9.70	3.6	–16.84	–2.57
	Bill length	0.80	–0.16	0.1	–0.43	0.10
	Sex	0.51	–1.02	1.9	–4.83	2.79
	Bill length*Sex	0.14	0.03	0.1	–0.11	0.17

Parameter likelihoods represent the weight of evidence that a parameter explains meaningful variation in the response variable (δ^15^N or δ^13^C values).

### δ^13^C

Birds with longer bills generally had more negative (terrestrial) δ^13^C values than those with shorter bills (Fig. 2b and 3b; AIC_c_ *w* of the random intercept model  =  0.66). Model selection indicated that both bill length and sex influenced δ^13^C values. The effect of bill length was greater than that of sex: bill length appeared alone in the top model, and models including this parameter received 80% of the support, while models including sex received only 50% of the support from the data (Table 2). However, weighted parameter estimates suggested that sex only weakly influences δ^13^C (Table 3; small estimate compared to standard error), while bill length has a slightly greater, but still small, impact.

## Discussion

We looked for evidence of local-scale niche segregation at 16 sites across the western sandpiper’s non-breeding range using stable-nitrogen and -carbon isotope analysis to infer aspects of diet composition and habitat use. Male and female western sandpipers have been hypothesised to occupy different foraging niches due to strong bill size dimorphism between the sexes [7], [9], [44], and indeed, quantitative measures of foraging modes vary with bill length [7], [9], [25], [26]. Our study is the first to contradict the hypothesis that strong bill size dimorphism between male and female western sandpipers leads to diet specialisation, using a test at a within-site level. We found that δ^15^N values of sandpiper whole blood did not vary according to either bill length or sex, providing little evidence that western sandpipers’ dietary composition is influenced by bill length. Based on differences in δ^13^C values, our results suggest that western sandpipers may exhibit slight differences in habitat use in relation to bill length, with more marine-influenced habitats used by birds with shorter bills and more freshwater-influenced habitats used by longer-billed birds. Differences in δ^13^C are driven more strongly by bill length than by sex per se.

Western sandpipers are partially latitudinally segregated by sex during the non-breeding season. We tested the hypothesis that local niche specialisation may be a mechanistic driver of this range-wide pattern. Dietary specialisation according to bill length at local scales is a key assumption of this mechanistic hypothesis. We did not find any evidence to suggest that bill length or sex affect δ^15^N in any predictable way within wintering sites, and our results fail to support the local niche specialisation hypothesis. In addition, we found that the relationship of bill length with latitude is sex-specific, as male, but not female, bills are longer at more southern latitudes. The ability of latitude to explain variation in bill lengths is very weak, even for males, and the significance of the relationship between bill length and latitude is strongly driven by sample size. However, predicted mean bill lengths for males at southern vs. northern range extremes differ by approximately 1 mm (Panama  =  23.15 mm, Humboldt Bay  =  22.10 mm), which may have biological significance for foraging behaviour.

Although diet does not differ according to bill length within wintering sites, the distribution of food may yet be important in influencing the non-breeding distribution of western sandpipers. Specialisation on different prey according to bill length, together with latitudinal patterns in the depth profile of the prey resource base has been proposed as a mechanism driving latitudinal segregation [6], [7], [9]. That individuals at more southern latitudes had longer bills, regardless of sex, has also been taken as evidence that the distribution of food resources influences latitudinal segregation of wintering sandpipers with respect to bill length [7], [32], particularly in light of the fact that prey become more deeply buried in the sediment at more southern latitudes [9]. Females, with their longer bills, may be more efficient foraging at southern latitudes where invertebrates are more deeply buried. Shorter-billed males may be more discerning about where they winter depending on whether they are short- or long-billed. Male and female western sandpipers may therefore choose to winter where they can forage most efficiently and are most competitive for food resources, even if a male and a female wintering together at the same site actually target and eat the same prey. A test of this version of the niche partitioning hypothesis (the global niche preference hypothesis) for the latitudinal segregation of the sexes requires measuring profitability of different prey types in relation to foraging mode, sex, and bill length.

Previously, diet partitioning between western sandpipers of different bill lengths has been inferred by observing foraging behaviour, particularly the proportion of pecking and probing foraging modes, and by exclosure and probe-mark experiments [7], [9], [25]–[27]. Little has been done to investigate the prey consumed by short- versus long-billed western sandpipers, either through visual observations (difficult for one of the smallest calidrid species), gut content analysis, or estimated using stable isotope analysis. One of the few studies to directly estimate diet of short- versus long-billed western sandpipers using stable isotope analysis found no difference in liver δ^15^N values between males and females at a migratory stopover site on the Pacific coast, nor any relationship between δ^15^N values and bill length [44]. Another study at the same stopover site found no relationship between the δ^15^N values of blood plasma and bill length in western sandpipers [45]. Thus, although short- and long-billed birds seem to favour different foraging modes, this does not appear to translate into any substantial differences in the respective trophic levels at which they forage, as inferred from δ^15^N values.

Sandpipers of different bill lengths may be eating similar prey, but using different foraging modes to access it. Alternatively, we may have incorrectly assumed that 1) use of the pecking versus probing foraging mode is correlated with the consumption of prey types that differ in trophic level; 2) bill length is strongly correlated with foraging mode; and 3) a mixed diet of both high and low trophic-level prey in different proportions according to bill length would lead to detectable differences in trophic level with bill size. High-quality video imagery similar to that used by Kuwae et al. [29], [30] might allow verification of Assumption 1 by providing a method for identifying the type of prey obtained by each foraging action. Verification of Assumption 2 requires foraging observations of individually-marked sandpipers across the non-breeding range. Previous work suggests that the proportion of peck vs. probe behaviour used by birds visually classified by an observer as male or female varies substantially between sites [7], [9]. A further complexity is added by the fact that the relationship between foraging behaviour and bill length may be far from as simple as males peck more while females probe more. In the only study to relate the bill length of previously captured and marked individuals to their foraging behaviour, Fernández and Lank ([26], see their Figure 1) found that males exhibit bimodal foraging behaviour, with some using predominantly pecking behaviour while others use predominantly probing behaviour. Considerable overlap with females might therefore be expected with respect to the type of prey selected. Verification of Assumption 3 requires prey isotope values at each site (isotopic baseline varies between locations) and use of an isotopic mixing model to more precisely determine how bill length influences consumption of different prey types [46]–[49]. Finally, it is possible that we did not detect any niche specialisation because all birds were captured in the same area, and we failed to sample the birds that were feeding in other habitats and on different prey. However, we believe this to be an unlikely scenario for the following reasons: 1) whole blood has a turnover rate of approximately one month, leading to ample time for birds to occupy a variety of habitats and feed on a diversity of prey, particularly given the dynamic nature of shorebird habitats due to fluctuating water levels; 2) where seasonal home range of western sandpipers has been determined from radio telemetry, individuals occupy an area of approximately 20 km^2^, where they are likely to encounter a variety of different microhabitats [50]; and 3) δ^13^C differed with bill length but δ^15^N did not, suggesting that we indeed captured birds foraging in a range of microhabitats.

Our failure to find any evidence that sexual segregation arises from dietary specialisation at the local scale suggests, despite the caveats above, that the influence of diet in explaining the latitudinal sex segregation of western sandpipers may require re-examination. Our findings, however, suggest a possible role for a factor that has received less attention in explaining latitudinal sex segregation: habitat. Niche partitioning by habitat segregation in shorebirds is well documented, with different species [51]–[53], sexes [14], [15], [19], [20], [54], and individuals occupying different foraging habitats according to morphology, their preferred foraging mode, or prey type. That male and female western sandpipers might segregate during the non-breeding season according to habitat is not a new idea, as a single study examining sex-specific habitat use at a wintering site found that females and males differentially used freshwater- vs. marine-influenced habitats [33]. Our results suggest that such habitat segregation may occur on a broader scale during the non-breeding season. Habitat preference might be bill length-dependent, if certain bill morphologies are better suited to foraging on particular substrate types [55], [56]. For example, longer-billed birds may prefer softer, muddier substrates that often tend to be found in areas with greater freshwater input (e.g. around estuaries and closer to shore). These softer sediments are more easily penetrated by probing actions, a foraging mode used more frequently by long-billed birds [57], [58]. If males and females tend to prefer and occupy different foraging habitats, then the broad patterns of latitudinal sex segregation observed in western sandpipers may arise due to latitudinal variation in habitat type and the associated differences in male-female sex ratio. A test of a habitat hypothesis requires characterising and quantifying general habitat qualities such as type, substrate, freshwater input, and more precisely assessing patterns of habitat use of males and females across their non-breeding range.

Alternatively, latitudinal segregation may stem from an interaction between habitat and variation in some other underlying ecological factor. Migratory western sandpipers show specific patterns of habitat use in relation to spatial gradients in danger and foraging habitat quality at stopover sites [59], [60]. Furthermore, evidence suggests that the non-breeding segregation of male and female western sandpipers may be partly explained by predation danger [8]. Latitudinal variation in predation danger across the non-breeding range together with a site’s particular danger-habitat quality profile could determine the sex-specific cost-benefit ratio of occupying different habitats, resulting in differential distribution of males and females both within sites and latitudinally. A test of this version of the predation danger hypothesis requires characterising and quantifying sex-specific patterns of habitat use in relation to the underlying spatial distribution of both food and predation danger. Our results indicate that females at some sites do preferentially occupy more freshwater-influenced habitats, which in a coastal environment may imply those habitats closer to shore as they are more strongly influenced by freshwater run-off. If food abundance is greater closer to shore [59], females may be more willing to trade off the benefits of greater food abundance with the costs of predation danger.

The factors influencing the non-breeding distribution of western sandpipers are by no means straightforward. The role of bill length and foraging niche in structuring the latitudinal distribution of male and female western sandpipers during the non-breeding season bears reconsideration, and other hypotheses, particularly ones that have the potential to lead to habitat segregation should be further explored and tested.
